# Structural Integrity Assessment of Concrete Sleepers by Modal Test Technique

**DOI:** 10.3390/ma16165614

**Published:** 2023-08-14

**Authors:** Jung-Youl Choi, Tae-Hyung Shin, Sun-Hee Kim, Jee-Seung Chung

**Affiliations:** 1 Department of Construction Engineering, Dongyang University, No. 145 Dongyangdae-ro, Punggi-eup, Yeongju-si 36040, Gyeongsangbuk-do, Republic of Korea; jychoi@dyu.ac.kr (J.-Y.C.); jschung@dyu.ac.kr (J.-S.C.); 2 Engineering HQ, Seoul Metro 5, Hyoryeong-ro, Seocho-gu, Seoul 06693, Republic of Korea; sss0930@seoulmetro.co.kr; 3 Department of Architectural Engineering, Gachon University, 1342 Seongnamdaero, Sujeong-gu, Seongnam-si 13120, Gyeonggi-do, Republic of Korea

**Keywords:** concrete sleeper, modal testing, numerical analysis, structural performance

## Abstract

Concrete sleepers used in railway engineering are subject to damage, such as cracks and breakage. Damaged concrete sleepers undergo changes to their material and structural properties, including response, mode shape, and natural frequency. Therefore, we have proposed modal testing in this study to quantitatively evaluate the structural integrity of concrete sleepers. The results of modal testing were compared with those of numerical analysis and visual inspection. In addition, an impact hammer test was conducted to evaluate the structural performance of damaged concrete sleepers. The results show that natural-frequency analysis using the modal-testing technique can usefully complement visual inspection for structural performance evaluation in the field.

## 1. Introduction

The dynamic response, natural frequency, mode shape, damping ratio, and other material and structural characteristics of concrete structures often change when the structure is damaged. Concrete sleepers are frequently damaged in the course of railway operation; poor materials and construction, manufacturing defects, environmental factors, repeated loads, and the driving characteristics of trains are among the internal and external factors responsible for concrete damage. 

Cracks in concrete sleepers are caused by structural and material factors. These cracks are enlarged by repeated loads of trains and exposure to the atmospheric environment. When exposed to the atmosphere, cracks expand owing to neutralization and freezing–thawing action, resulting in damage to track components [1].

**Figure 1:**
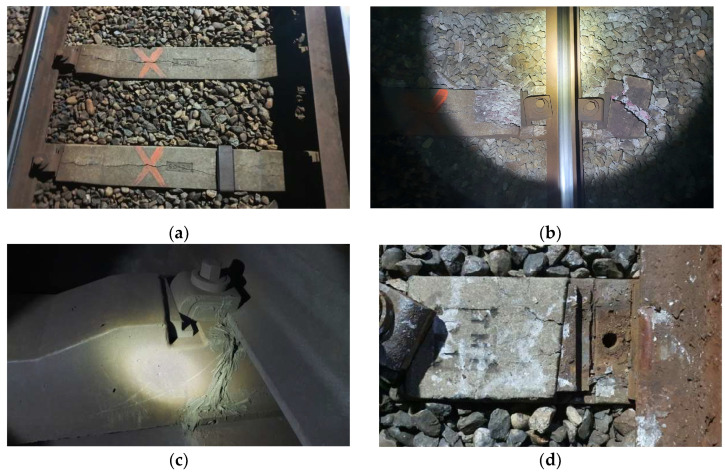
Photographs of various damaged concrete sleepers. (**a**) Longitudinal cracking; (**b**) damage around fasteners; (**c**) cracking of rail seat; and (**d**) cracks near insert.

According to South Korean standards, damaged sleepers (Figure 1) are identified by visual inspection and repaired or replaced based on the subjective judgment of the inspector, as shown in Figure 1. Such qualitative evaluations of structural integrity have clear limitations [2]. 

In the case of sleepers with axial cracks and with resilience pads attached to the lower part, the lateral resistance is reduced in the curved rail. Excessive lateral pressure occurs owing to poor contact between the bottom of the sleeper and the ballast because of the cant imbalance while the train is running. Such excessive lateral pressure generates high stress in the axial direction of the sleeper as the stress is concentrated in the structurally weak landfill in the sleeper [1].

In the track-facility performance evaluation of South Korea [2], shown in Table 1, sleepers were assessed through visual inspection (on basic tasks) and the rebound hardness test (on optional tasks). Visual inspection is a qualitative evaluation of sleeper damage based on the subjective judgment of the inspector. The rebound hardness test is a quantitative evaluation of the material strength of the concrete; it is not a test to evaluate the structural integrity of damaged sleepers.

In operating urban passenger lines, concrete sleepers can only be visually inspected at night (i.e., after the passenger-train operation has ended). This limits the working time to ~3 h or less, making it difficult in practice to evaluate the condition of the numerous sleepers in the field.

Indoor and outdoor experiments have been conducted in South Korea and overseas to study damage to sleepers.

Choi [3] analyzed changes to the dynamic characteristics of concrete sleepers using the modal-testing technique to evaluate the damage. A numerical model of the conditions of each track component was used to confirm analytically the experimentally observed effects of rail-fastening–hole loosening and damage on the structural characteristics of concrete sleepers.

Remennikov and Kaewunruen [4] analyzed the effect of boundary conditions on the natural frequency and damping ratio of concrete sleepers. Concrete sleepers were tested using the percussive hammer excitation technique in the frequency range of interest, 0–1600 Hz. Further, frequency response functions (FRFs) were measured using the Bruel & Kjaer PULSE dynamic analyzer. The results showed that the vibration parameters of concrete sleepers are necessary for the development of a realistic dynamic model of railway tracks that can predict the response to impact loads owing to wheel burn and rail irregularities.

Kaewunruen and Remennikov [5,6,7,8] investigated the dynamic characteristics of railroad tracks through experiment and numerical analysis using the impact excitation method. The sensitivity effect on the free-vibration characteristics of concrete sleepers in the field caused by changes in the stiffness and material properties of the rail pads was analyzed. Timoshenko beam and spring elements were used in the finite element analysis. The finite element analysis results confirmed that the rail pad parameters have a nonlinear effect on the effective stiffness affecting the field track system and can significantly affect the frequency and mode shape of additional modes. The frequency response function (FRF) was investigated in the frequency range of 0–1600 Hz using a Bruel & Kjaer PULSE vibration analyzer. The dynamic characteristics of the track site with good conditions confirmed that the stiffness of the pad and ballast was in a narrow range of 800–1500 MN/m for the rail pad and 150–470 MN/m for the ballast. 

Taherinezhad et al. [9] investigated the behavior of prestressed concrete sleepers and found that they should meet the durability requirements of concrete sleepers. Further, the quantification of dynamic loads and the structural behavior of sleepers, tracks, and their interactions with other components of the failure mechanism were generated.

You and Kaewunruen [10] evaluated the residual fatigue life of concrete sleepers based on field-loading conditions. The field-loading conditions, time-dependent dynamic properties, and dynamic bending moments of prestressed concrete sleepers were quantitatively analyzed. Consequently, it was confirmed that concrete sleepers can generally suffer from a dynamic fatigue load spectrum over their entire lifetime, and simultaneously, the static and dynamic load-bearing capacity of concrete sleepers deteriorates over time.

You et al. [11] confirmed the structural behavior of railway prestressed concrete sleepers with rail sheet wear through nonlinear analysis. Based on the finite element analysis of the prestressed concrete sleeper with abrasion on the rail sheet, it was confirmed that the theoretical analysis and experimental results were similar.

Kaewunruen and Remennikov [12] confirmed the effect of asymmetric wheel load on the flexural response and failure of railway concrete sleepers through analysis and experimentation. Based on the experiment, it was confirmed that the tensile stress on the upper surface of the concrete sleeper tends to decrease under high wheel impact load and is redistributed to significantly increase in the lower part of the high-load rail sheet.

Ferdous et al. [13] analyzed damage to concrete sleepers owing to the degradation of track materials, axial cracks in sleepers, and impact loads during train operation. Through indoor testing and numerical analysis, Silva et al. [14] verified that the main defects in prestressed concrete sleepers occur as damage directly under the rail. Tatarinov et al. [15] found two main causes of damage to prestressed concrete sleepers: dynamic loads and exposure to the atmosphere. They visually inspected and analyzed the state of cracks in the sleepers in the field through ultrasonic flaw detection.

Choi et al. [1] analyzed the types of damage to concrete sleepers installed in operational sharp-curve sections; they found mainly axial cracks. They also confirmed that the stress in the sleepers could be reduced by replacing aged gravel.

Lee et al. [16] experimentally confirmed the applicability of shear wave tomography as a destructive inspection method for high-speed railway concrete track slabs. The layer separation map created through shear wave tomography could clearly identify the location of the defect.

Most of the non-destructive testing studies on concrete sleepers were conducted by Remennikov and Kaewunruen. Based on the research by Remennikov and Kaewunruen, the structural integrity of concrete sleepers was confirmed through non-destructive testing of sleepers installed in Korea.

In South Korea, most of the experimental and numerical research has been conducted on the cracks in concrete sleepers, however, there exists no research on crack integrity through non-destructive testing. This study focuses on post-tensioned concrete sleepers, which are buried in gravel or concrete tracks; in this case, only the surfaces of the sleepers can be visually inspected. Depending on the type of track, it is buried in a gravel or concrete roadbed; thus, only the upper surface of the concrete sleeper can be inspected. Consequently, the condition evaluation grade of the sleeper is determined based on the condition of the upper surface.

In the structure damage detection method using changes in dynamic characteristics of structures when a structure changes physically, such as appearance of cracks, the dynamic characteristics of the structure also change.

In this study, we conducted an impact hammer test, which is a portable non-destructive test, to evaluate more quantitatively the structural integrity of 20 damaged concrete sleepers in the field and analyzed the correlation between damage and changes in dynamic characteristics.

## 2. Visual Inspection of Damaged Concrete Sleepers

**Figure 2:**
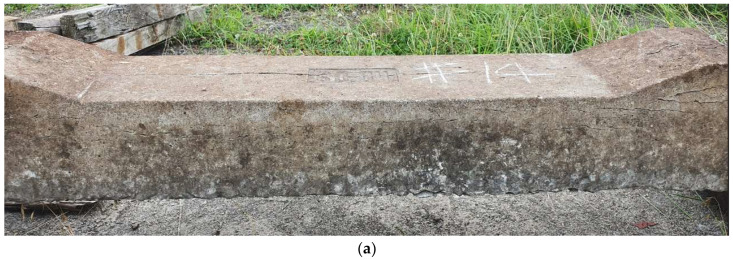

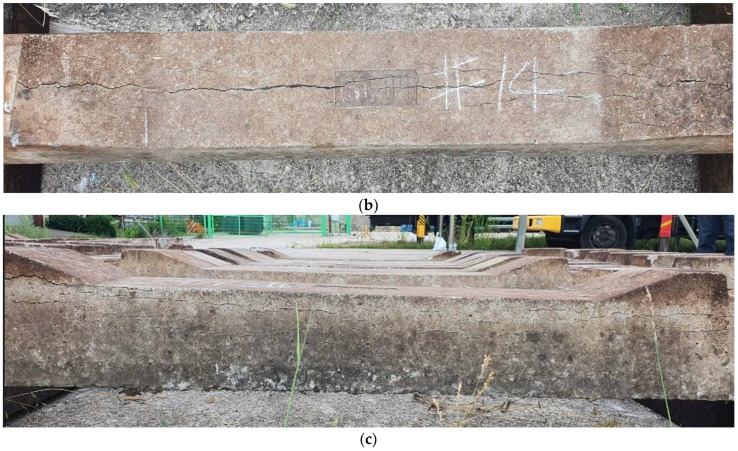
Visual inspection (crack check). (**a**) Front; (**b**) top; and (**c**) backward.

**Figure 3:**
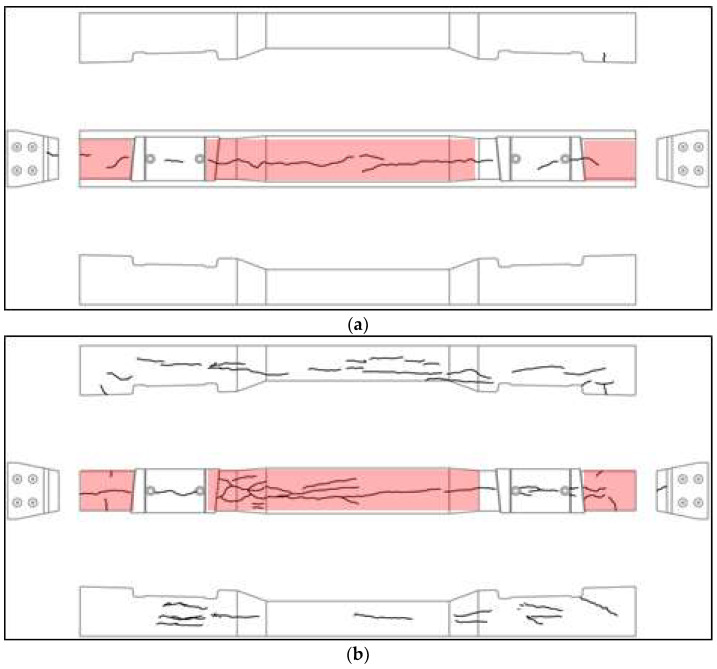

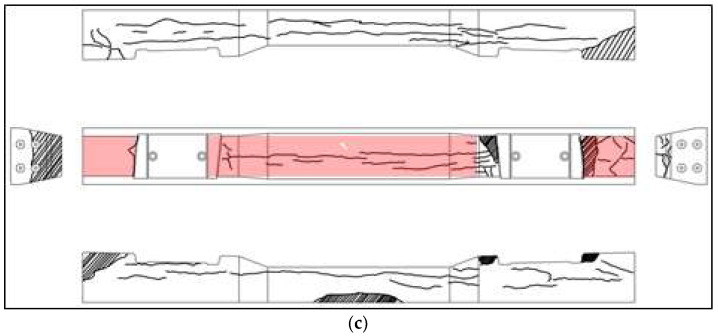
Examples of visual inspection (crack check) of various sleepers. (**a**) #5; (**b**) #13; and (**c**) #18.

We conducted on-site visual inspections of 20 concrete sleepers that were designated for replacement because of damage. Figure 2 and Figure 3 show the damaged concrete sleepers and conditions of the sleeper cracks.

In sleeper #5 shown in Figure 3a, an axial crack penetrated the area around the fastening-hole insert and center of the sleeper. The top surface of sleeper #13 was similar to that of #5, as shown in Figure 3b, but many cracks were also found on the sides. Sleeper #18 was similar to #13 in terms of top-surface condition, except for the locally damaged shoulder, as shown in Figure 3c. However, damage to the side of the sleeper was relatively severe.

In a visual inspection at night, the inspector would only be able to observe the condition of the top surface and would thus judge #13 and #5 to be similar. Moreover, although the cracks in the axial direction were similar in all three sleepers, #18, which has more damaged areas, may be evaluated as being in the worst state.

## 3. Impact Hammer Testing of Damaged Concrete Sleepers

### 3.1. Measurement System Overview

**Figure 4:**
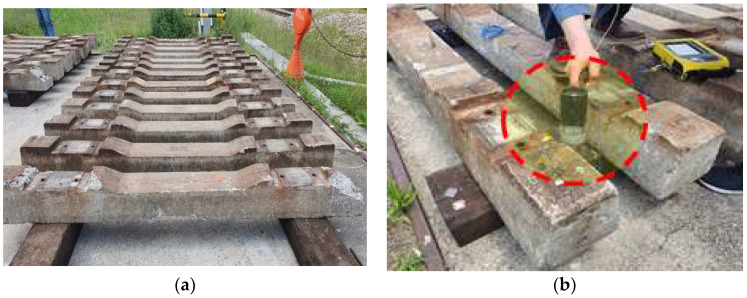
Photographs of the modal test using an impact hammer. (**a**) Damaged concrete sleepers; (**b**) impact hammer test.

We conducted modal tests on damaged concrete-sleeper specimens collected from the operating line, as shown in Figure 4a. In the impact hammer test, it is possible to check the ballast damage by exciting the sleeper installed on the site with an impact hammer. We performed an impact hammer test to measure the dynamic response (acceleration) compared to the impact load. As shown in Figure 4b, an accelerometer was installed on the surface of the sleeper to measure the vertical acceleration occurring when the impact hammer struck. The sensitivities of the measuring system sensors are summarized in Table 2.

### 3.2. Result of Each Sleeper Frequency Response Function Calculation

The FRF is the ratio of dynamic response to impact load; it allows us to calculate the characteristics of dynamic responses in the frequency domain and the relationship between mass and stiffness. Through FRF, values can be estimated in the range below the natural frequency in the case of dynamic stiffness and the range exceeding the natural frequency in the case of dynamic mass.

Two frequency bands occurred in all sleepers when excited with an impact hammer.

**Figure 5:**
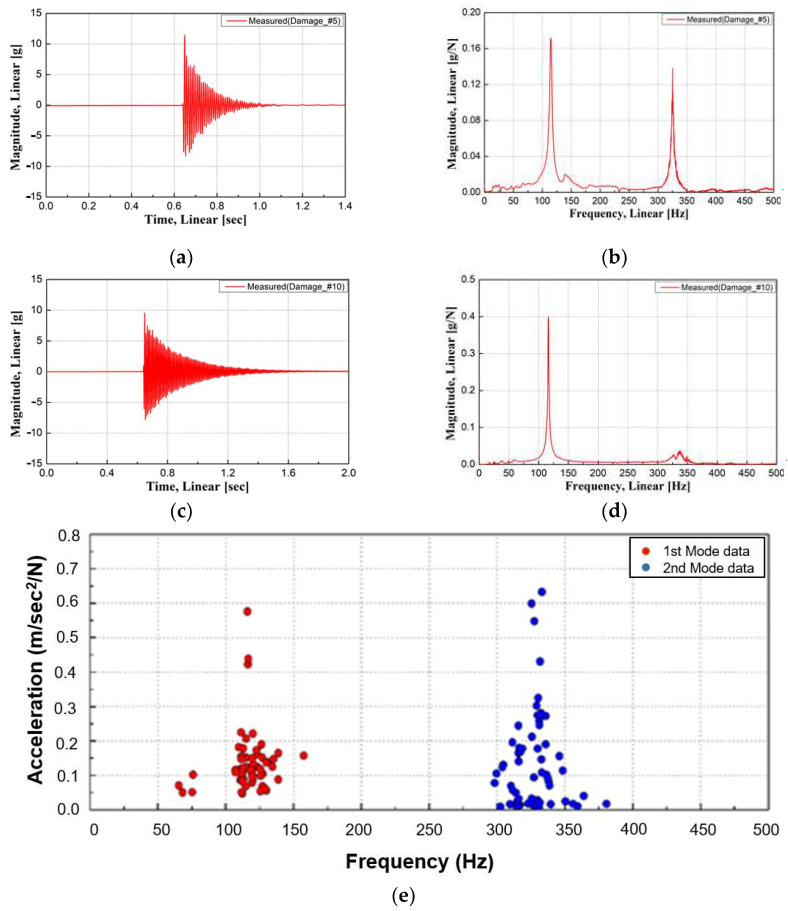
Measurement results of frequency response function (FRF): (**a**) time–acceleration for #5; (**b**) FRF for sleeper #5; (**c**) time–acceleration #10; (**d**) FRF for sleeper #10; and (**e**) distribution of FRF results for all sleepers.

As shown in Figure 5a,c, we measured the load when the hammer struck and the acceleration over time. We calculated the natural frequencies according to the frequency response function (FRF) of the sleeper, as shown in Figure 5b,d.

Based on the FRF analysis results, the measured natural frequencies of the 20 damaged concrete sleepers were divided into first and second modes, as shown in Figure 5e. The first-mode frequency of most sleepers exceeded 100 Hz, but in some cases, the natural frequency was at half the level of the others. The second-mode frequency was in the range of 298.28–380.63 Hz.

**Figure 6:**
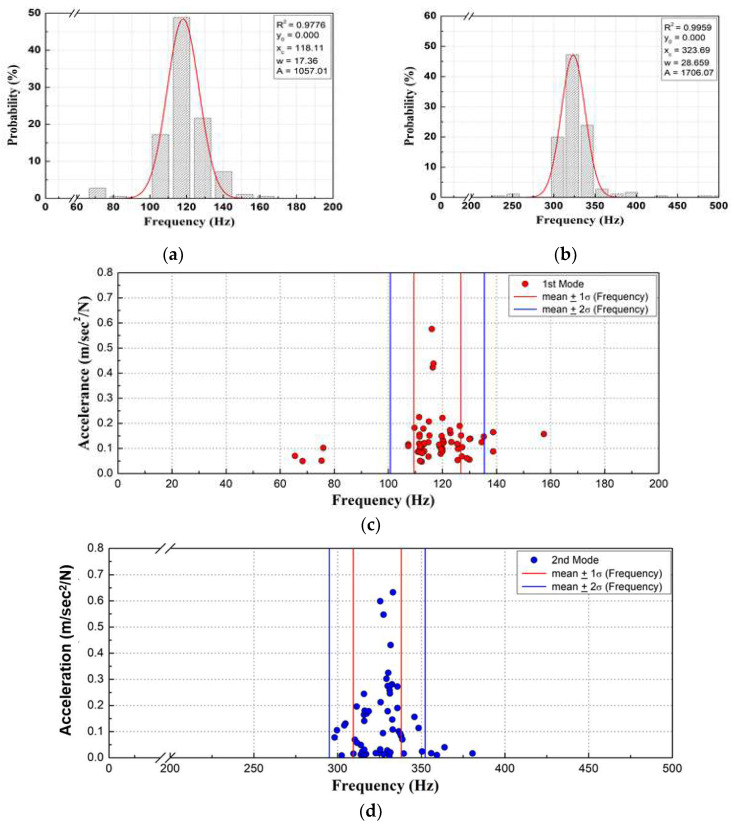
Analysis of measured frequency response function for damaged concrete sleepers. (**a**) Gaussian graphs of first mode; (**b**) Gaussian graphs of second mode; (**c**) frequency ranges first mode; and (**d**) frequency range of second mode.

We performed a Gaussian probability density analysis using the measured natural frequencies of each mode, as shown in Figure 6a,b. The distribution of the FRF for the damaged sleepers had a high correlation coefficient and small standard deviation, exhibiting the features of a normal distribution. We calculated the average probability and standard deviation for each mode. As shown in Figure 6c,d, most of the measurements were distributed within the 2σ range, and the natural frequencies of some sleepers were relatively low in the first mode.

**Figure 7:**
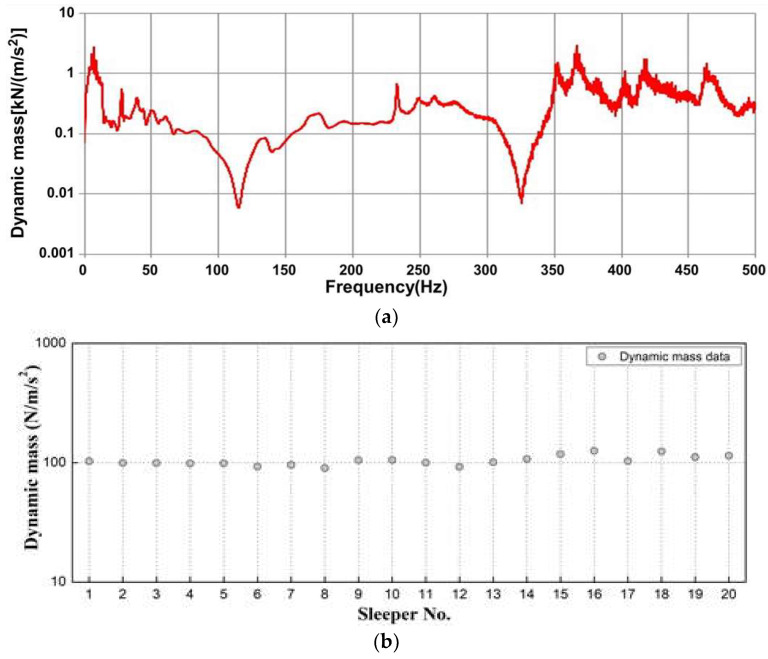
Dynamic mass estimation results. (**a**) Results for #5; (**b**) result for all sleepers using FRF.

Figure 7a shows the effect of concrete-sleeper damage on dynamic mass. By estimating the dynamic mass according to damage to concrete sleepers, the dynamic mass results from 20 sleepers were calculated at 0.1 kN/m/s^2^. Figure 7b shows that the dynamic mass was constant for all sleepers and that the effect of damage was minimal.

**Figure 8:**
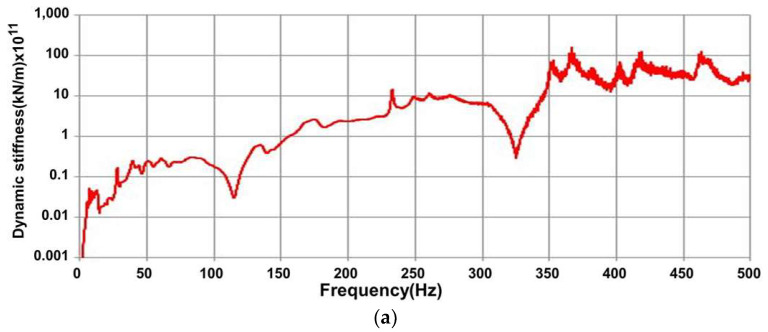

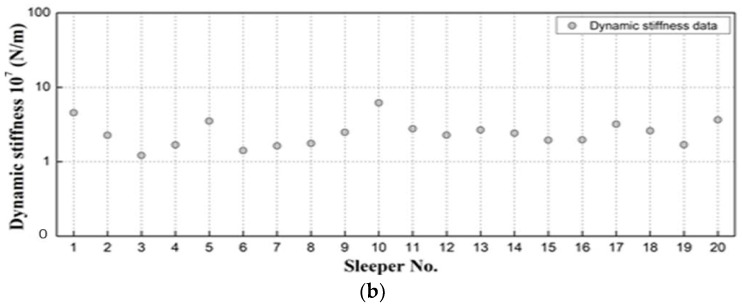
Dynamic stiffness estimation results. (**a**) Results for #5; (**b**) result for all sleepers using FRF.

Figure 8a,b show the effect of concrete sleeper damage on dynamic stiffness and dynamic stiffness of all the sleepers as calculated using the FRF: it ranged from 1.22 to 6.18 (10^7^ N/m) and the damage to the concrete sleepers directly affected it. Contrarily, the dynamic mass was constant for each sleeper as shown in Figure 7b, and the effect of the damage was relatively small. Therefore, we confirmed that the dynamic stiffness decreased because of damage to the concrete sleepers, leading to a decrease in their natural frequency [17].

In the case of low-damping structures whose amplitude decreased exponentially with time, the damping ratio of the structure over time in the free-vibration region can be estimated as shown in Equation (1) [17].

(1) $$\delta =ln\left(\frac{u_{1}}{u_{2}}\right)=\frac{2\pi \xi }{\sqrt{1-\xi^{2}}}≅2\pi \xi ,$$

where, δ represents the logarithmic decrement, that is, the reduction ratio of the amplitude after one cycle (T_d_) of free vibration. If the damping ratio is small (ξ<0.2), it can be approximated as $\sqrt{1-\xi^{2}}>0.9798\approx 1$.

**Figure 9:**
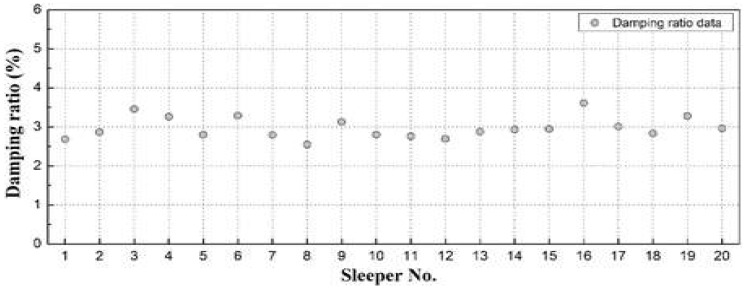
Measured damping ratios of all sleepers.

We calculated the damping ratio in the free-vibration region for each sleeper, as shown in Figure 9. Although the ratio showed some variation, it remained within 3 ± 0.5%, the material damping level of concrete, indicating that the damage did not directly affect it [10].

## 4. Numerical Analysis

The natural frequencies of the concrete sleepers were analyzed numerically using ANSYS Workbench Ver. 2021 R2 [18], a general-purpose structural analysis program. Numerical analysis is the process of numerically approximating a value using a computer to obtain an approximate value. In this study, numerical analysis can confirm the behavior of a structure through an approximate solution without performing an experiment as long as there are only material properties.

**Figure 10:**
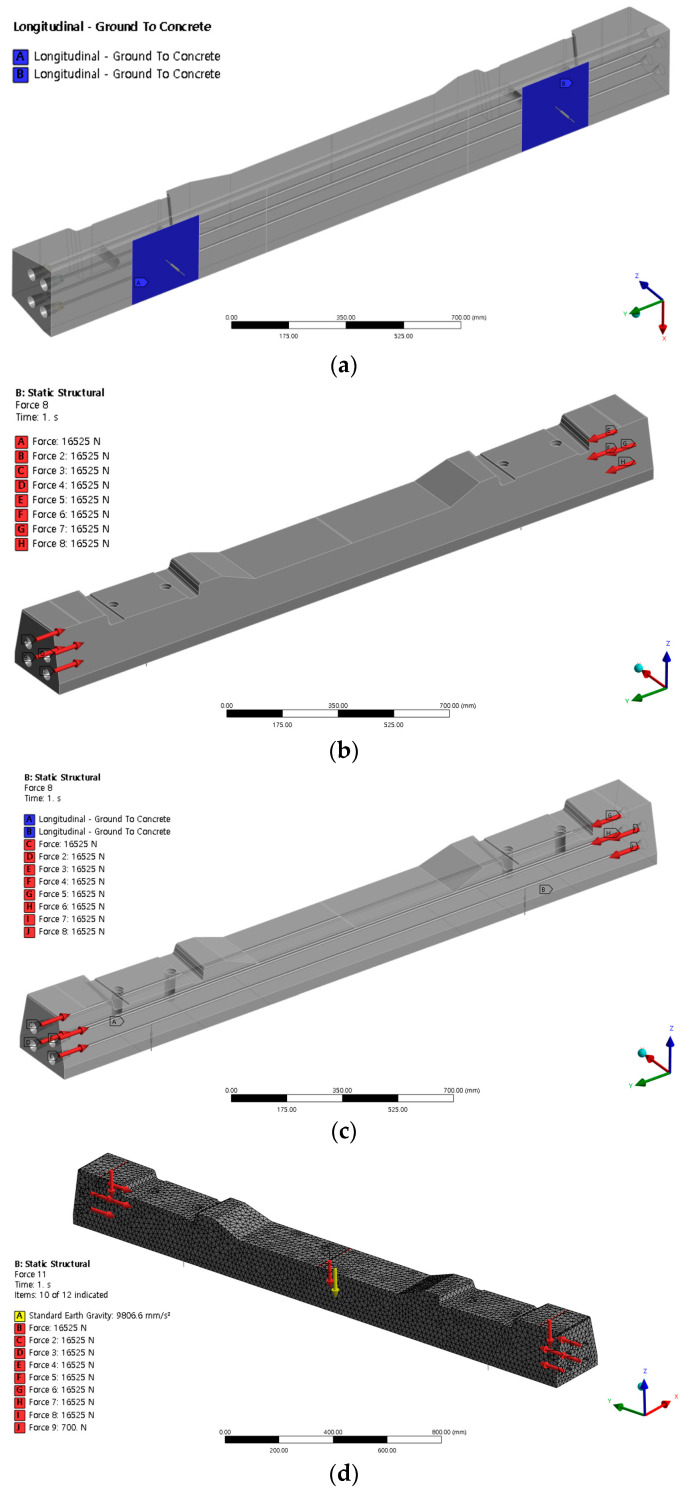
Loads and boundary conditions. (**a**) Boundary conditions; (**b**) prestress; (**c**) modal analysis; and (**d**) post-tensioning concrete sleeper modeling.

Nodes and elements were calculated automatically during their generation in the ANSYS Workbench program. The mesh of the sleeper contained 242,085 nodes and 144,779 elements. The element size of the sleeper was assumed to be 20 mm. In the numerical analysis, we modeled both normal and damaged sleepers under the impact load (700 N) and boundary conditions used in the modal test. Additionally, as shown in Figure 10, we simulated the internal steel bars of the post-tensioned sleepers and introduced tension to each steel bar. For the boundary conditions, we applied the compression spring stiffness (200 kN/mm) of the wooden sleepers that supported the ends of the concrete [17]. Table 3 lists the specifics of the finite element model. 

**Figure 11:**
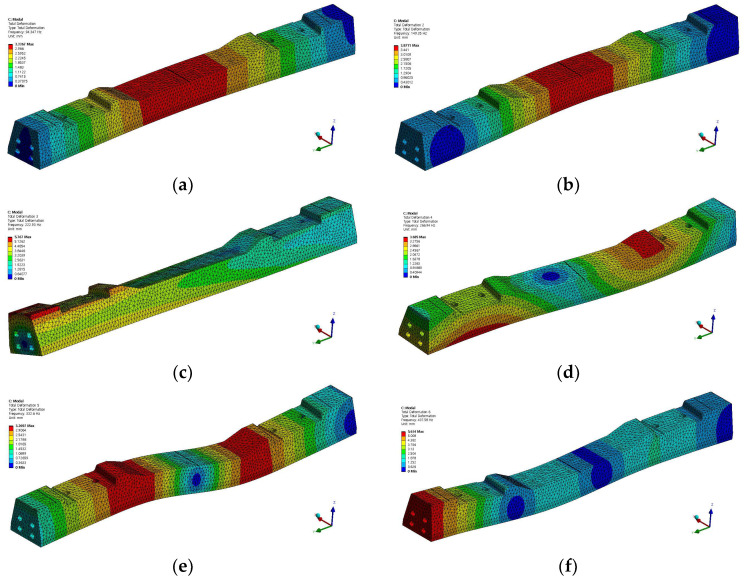
Analysis results (mode shape). (**a**) 1st mode (94.34 Hz); (**b**) 2nd mode (149.35 Hz); (**c**) 3rd mode (222.93 Hz); (**d**) 4th mode (266.94 Hz); (**e**) 5th mode (332.6 Hz); and (**f**) 6th mode (437.58 Hz).

The mode shapes and corresponding frequencies of the concrete sleepers are shown in Figure 11. The first mode appeared at 94 Hz as shown in Figure 11a, and deformation occurred in the longitudinal direction, which is the direction of the train. The secondary mode appeared at 149 Hz as shown in Figure 11b. The third mode appeared at 222 Hz as shown in Figure 11c and had sleeper rotation characteristics. The fourth mode appeared at 266 Hz as shown in Figure 11d and had longitudinal deformation characteristics and an inflection point. The fifth mode appeared at 332 Hz as shown in Figure 11e and had the bending deformation characteristics of sleepers and an inflection point. The sixth mode is shown in Figure 11f and appeared at 437 Hz.

**Figure 12:**
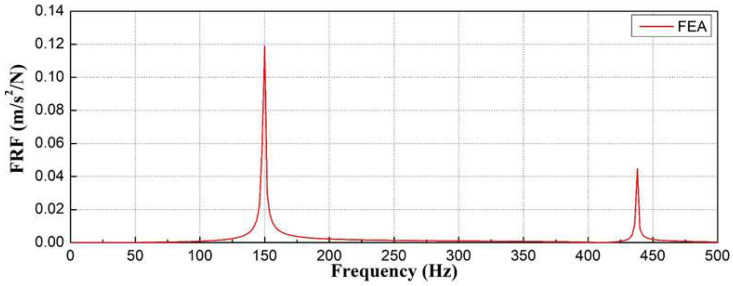
Natural frequencies of concrete sleeper in normal conditions according to finite element analysis.

As shown in Figure 12, the natural frequencies at which deformation occurred in the concrete sleepers owing to the bending mode were found to be 149.35 Hz in the second mode and 437.58 Hz in the sixth mode.

## 5. Correlation between Damage to the Concrete Sleeper and Natural Frequency

After comparing and analyzing the natural frequencies of the damaged concrete sleepers, a modal test was performed on the damaged concrete sleepers in the field.

**Figure 13:**
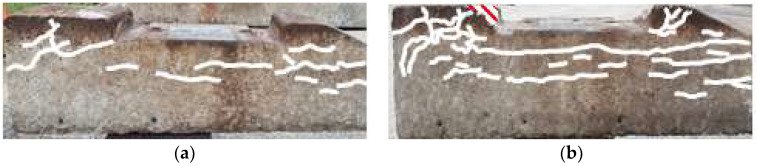
Visual inspection (crack check) of the sleepers used for comparison of modal test and numerical analysis results. (**a**) Crack check of #1; (**b**) crack check of #2.

We compared the analytical and measured natural frequencies of two concrete sleepers whose crack propagation patterns appear to have similar damage states (as shown in Figure 13).

Among the twenty damaged concrete sleepers, the numerical analysis and field measurement results were compared and analyzed for two concrete sleepers evaluated similarly to the exterior survey results. 

**Figure 14:**
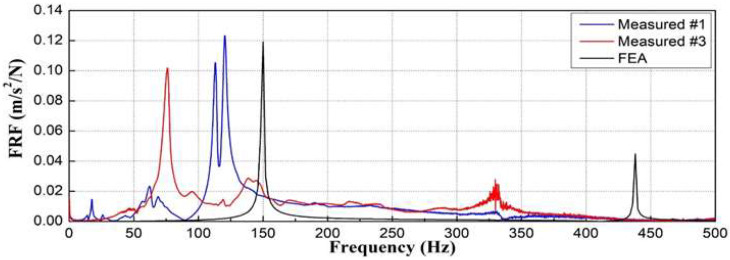
Comparison of modal test and numerical analysis results for two visually similar sleepers.

As shown in Figure 14, the measured natural frequencies of the sleepers differed significantly: 120.47 and 75.94 Hz. They were also, respectively, 20% and 49% smaller than the analytical natural frequency under normal conditions (149.358 Hz). 

In the variable affecting the natural frequency of the structure, the change in dynamic mass owing to damage to concrete sleepers was negligible. Furthermore, as the change in dynamic stiffness was clear, the decrease in dynamic stiffness according to the degree of damage to the sleeper affected the structural integrity of the concrete sleeper. 

This confirms the need for quantitative condition assessments based on natural frequencies, as the structural integrity of sleepers can differ even with visually similar damage.

**Figure 15:**
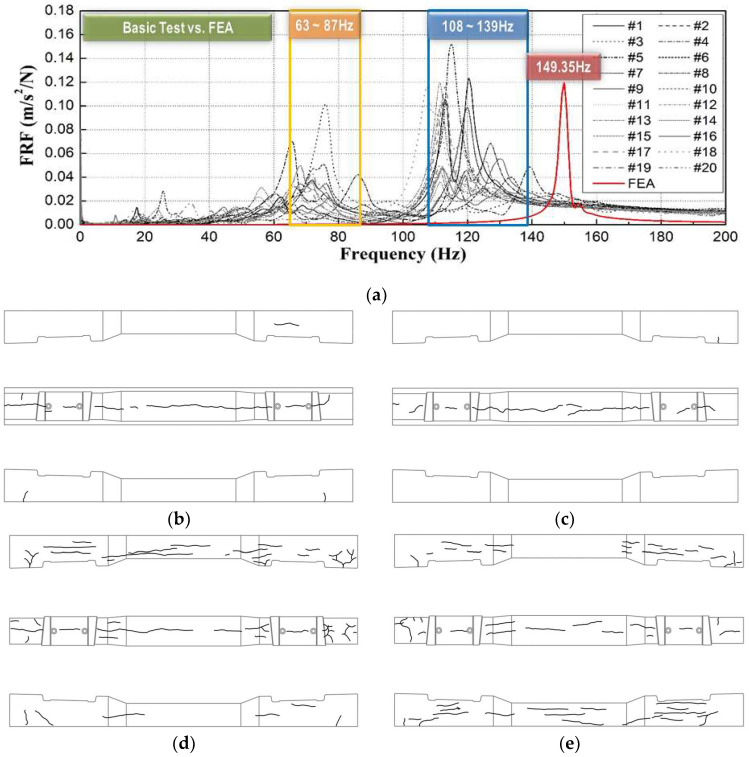
Comparison of modal test, numerical analysis, and visual inspection results. (**a**) Measured and analyzed natural frequency; (**b**) #6 (75.31 Hz); (**c**) #5 (115.16 Hz); (**d**) #15 (68.28 Hz); and (**e**) #16 (112.34 Hz).

Similar comparisons for 20 damaged concrete sleepers (Figure 15) show that the measured natural frequencies were smaller than those from numerical analysis and could be broadly categorized into two frequency bands.

As shown in Figure 15, the measured natural frequencies of sleepers #6 and #5 and #15 and #16, which had similar surface conditions, significantly differed. Interestingly, #15, which was classified as more severely damaged, showed a similar measured natural frequency to #6. This again indicates that it is inappropriate to evaluate the structural integrity of sleepers only by their surface condition.

The structural integrity of the #3 sleeper with cracks in the axial direction of the concrete sleeper was evaluated as lower than that of the #5 sleeper. However, in the case of the natural frequency measured in the field, the natural frequency was small in the #3 concrete sleeper with cracks on the side of the concrete sleeper. During the field investigation, concrete sleepers were embedded in gravel or concrete because of the nature of the track structure, making it difficult to identify cracks on the side and bottom.

During the field investigation, concrete sleepers were embedded in gravel or concrete because of the nature of the track structure, making it difficult to identify cracks on the side and bottom and accurately investigate the state of damage. In the concrete sleeper of #3, where cracks also occurred on the top, directly below the rail, and on the side of the concrete sleeper, the dynamic stiffness decreased and the natural frequency was measured to be small.

Thus, it is inappropriate to evaluate the structural integrity of concrete sleepers by visual inspection alone, particularly considering that a detailed cross-section inspection of sleepers is impossible during night-time inspections on operational lines.

## 6. Conclusions

This study proposed impact hammer testing, a portable technique that can be easily applied in the field for quantitative assessment of the structural integrity of concrete sleepers. We proved the validity of the modal-testing approach by comparing the results of field testing, numerical analysis, and visual inspection. The results are as follows:

1. For 20 damaged concrete sleepers, the measured natural frequencies obtained using the impact hammer test were approximately 42–93% of that predicted for normal conditions by numerical analysis (149.35 Hz). According to the FRF-based estimation results, the change in dynamic mass caused by the damage to concrete sleepers was negligible, whereas dynamic stiffness significantly differed among sleepers. Furthermore, the measured damping ratio of each sleeper was 3 ± 0.5%, which is the material damping level of concrete; this indicates that the effect of sleeper damage on the damping ratio was small.
2. A comparative analysis of the modal-testing, analytical, and visual-inspection results showed that the measured natural frequencies of the two concrete sleepers classified as similarly damaged in the visual inspection were, respectively, 20 and 49% smaller than the calculated natural frequency under normal conditions. This experimentally and analytically demonstrates the limitations of evaluating the structural integrity of concrete sleepers solely by their visual state.
3. Natural-frequency analysis through modal testing can be used for quantitatively evaluating the structural integrity of many sleepers easily in a short time. Therefore, considering the difficulty of night-time inspections on operational lines, the proposed modal-testing method provides a complementary method of visual-inspection-based sleeper-condition assessment in the field.

## Figures and Tables

**Table 1:** Criteria of performance evaluation items for the concrete sleeper.

Description	Evaluation Items	Methodology	Concrete Sleeper			A	B
			Pre-Stressed Concrete	Reinforced Concrete Sleeper			
				E Type	A Type		
Safety	Surface crack, defect, shoulder condition	Visual inspection	Entire			O	
	Pre-stressed exposed	Visual inspection	Entire			O	
	Rebound hardness test	Non-destructive test	3 sections/500 m				O
Durability	Accumulated tonnage, velocity, used year	Maintenance data	Entire			O	
	Rebound hardness test	Non-destructive test	3 sections/500 m				O
	Depth of carbonation	Field test	1 section/500 m				O
	Surface crack	Visual inspection	Entire			O	

E type: Embedded sleeper type; A type: anti-vibration type; A: compulsory items; and B: elective items.

**Table 2:** Sensitivity of sensors for impact hammer test.

Sensor Type	Impact Hammer	Accelerometer		
Channel	Ch. 1	Ch. 2	Ch. 3	Ch. 4
Sensitivity	0.2301 mV/N	1024.0 mV/g	1018.0 mV/g	993.2 mV/g

**Table 3:** Properties of finite element model.

Items	Properties	
Sleeper type	Post-tensioning Sleeper	
Sleeper size (mm)	212 × 240 × 2300	
Compressive strength (MPa)	59	
Post-tensioning steel bar	Tensile strength (MPa)	1230
	Diameter (mm)	Ø9.2
	Length (mm)	2240
Post-tensioning force (kN/EA)	66.1	
Sleeper supporting condition (spring stiffness)	Wooden sleeper supporting condition (200 kN/mm)	

## Data Availability

Not applicable.
